# Pregabalin-Induced Bullous Pemphigoid: A Case Report of a Rare Drug-Triggered Autoimmune Skin Disorder

**DOI:** 10.7759/cureus.69649

**Published:** 2024-09-18

**Authors:** Rachel Tovar, Daniel T Jones, Ramaditya Srinivasmurthy, Meghana Pandit, Liawaty Ho

**Affiliations:** 1 Internal Medicine, Touro University Nevada College of Osteopathic Medicine, Henderson, USA; 2 Oncology, Comprehensive Cancer Centers of Nevada, Las Vegas, USA

**Keywords:** topical corticosteroids, pregabalin, drug-induced bullous diseases, bullous pemphigoid, autoimmune skin disorder

## Abstract

Bullous pemphigoid (BP) is a common autoimmune blistering disorder primarily affecting the elderly, characterized by intense pruritus and tense bullae on the skin. We report the case of a 75-year-old female with a history of breast cancer who developed BP on both feet following the initiation of pregabalin for pain management. Histopathological examination confirmed BP, and symptoms improved with topical corticosteroid treatment and discontinuation of pregabalin. This case highlights the potential of pregabalin to induce BP and underscores the importance of recognizing medication-induced bullous diseases for prompt diagnosis and management.

## Introduction

Bullous pemphigoid (BP) is a common pruritic bullous skin disease primarily affecting older adults aged 60 to 80 years [1]. It is an autoimmune disorder characterized by the formation of tense blisters and bullae on the skin, often following a prodromal phase of intense itching and urticaria or papular lesions [2]. Bullous pemphigoid shows a higher prevalence in individuals with specific genetic predispositions and is slightly more common in women than in men [3]. The pathogenesis of BP involves an autoimmune response against BP180 (type XVII collagen) and BP230, two structural proteins in the epidermal basement membrane critical for epidermis-dermis adhesion. Autoantibodies targeting BP180 and BP230 lead to the activation of the complement system and recruitment of inflammatory cells to the dermal-epidermal junction. This inflammatory response causes the separation of the epidermis from the dermis, resulting in subepidermal blister formation [3].

The exact cause of BP is not fully understood, but it is believed to result from a combination of genetic, environmental, and immune factors. Several drugs, such as diuretics, non-steroidal anti-inflammatory drugs (NSAIDs), antibiotics like amoxicillin and ciprofloxacin, tumor necrosis factor (TNF) inhibitors, anticonvulsants, and dipeptidyl peptidase-4 (DPP-4) inhibitors, have been associated with the development of BP [3]. These medications are thought to influence the immunological mechanisms by modulating calcium channel function, which may inadvertently enhance immune dysregulation and trigger autoantibody production against BP180 and BP230 [3]. Additionally, neurological disorders (e.g., Alzheimer’s disease, Parkinson's disease, multiple sclerosis) and physical factors such as trauma or radiation therapy can also contribute to the onset of BP [3].

The diagnosis of BP involves a combination of clinical evaluation, histopathological examination, and immunopathological testing. Direct immunofluorescence (DIF) of a perilesional skin biopsy is the gold standard for diagnosis, revealing linear deposits of IgG and C3 along the basement membrane zone [3]. Serological tests for circulating autoantibodies against BP180 and BP230 can further support the diagnosis. Treatment centers on reducing inflammation, suppressing the immune response, and effectively managing symptoms. First-line therapies often include topical and systemic corticosteroids. For patients who do not respond to corticosteroids or have significant side effects or comorbidities like severe osteoporosis or diabetes, immunosuppressive agents such as mycophenolate mofetil, methotrexate, and azathioprine may be prescribed. In refractory cases, treatments like intravenous immunoglobulin (IVIG), rituximab, or omalizumab can be considered [4]. Early recognition and appropriate management of BP are crucial to prevent complications and improve the quality of life for affected patients.

Pregabalin, marketed under the brand name Lyrica, is an anticonvulsant and analgesic medication primarily used to treat neuropathic pain, fibromyalgia, and generalized anxiety disorder. It functions by binding to the alpha-2-delta subunit of voltage-gated calcium channels in the central nervous system, inhibiting the release of excitatory neurotransmitters such as glutamate, norepinephrine, and substance P [5]. Despite its therapeutic benefits, pregabalin has been associated with various adverse effects, including dizziness, somnolence, and peripheral edema. In the United States, pregabalin is widely used, with millions of prescriptions written annually; in 2020 alone, over 6.4 million people used pregabalin [6]. 

Drug-induced BP is relatively rare, and cases specifically linked to pregabalin are even less common. This case report highlights a rare instance of pregabalin-induced bullous pemphigoid, emphasizing the importance of recognizing drug-induced etiologies early in patients presenting with new or unusual dermatologic symptoms. 

## Case presentation

A 75-year-old female presented to the oncology office following a biopsy that confirmed well-differentiated invasive ductal carcinoma with insufficient material to assess ER, PR, and HER-2 status. She completed accelerated partial breast irradiation (APBI) on June 29, 2018. Follow-up visits through October 2018 and subsequent years showed no new breast masses or significant findings, with regular surveillance and benign imaging results. The patient reported no new complaints other than an extensive cardiac history and obstructive sleep apnea (OSA), managed by cardiology and pulmonology.

**Figure 1:**
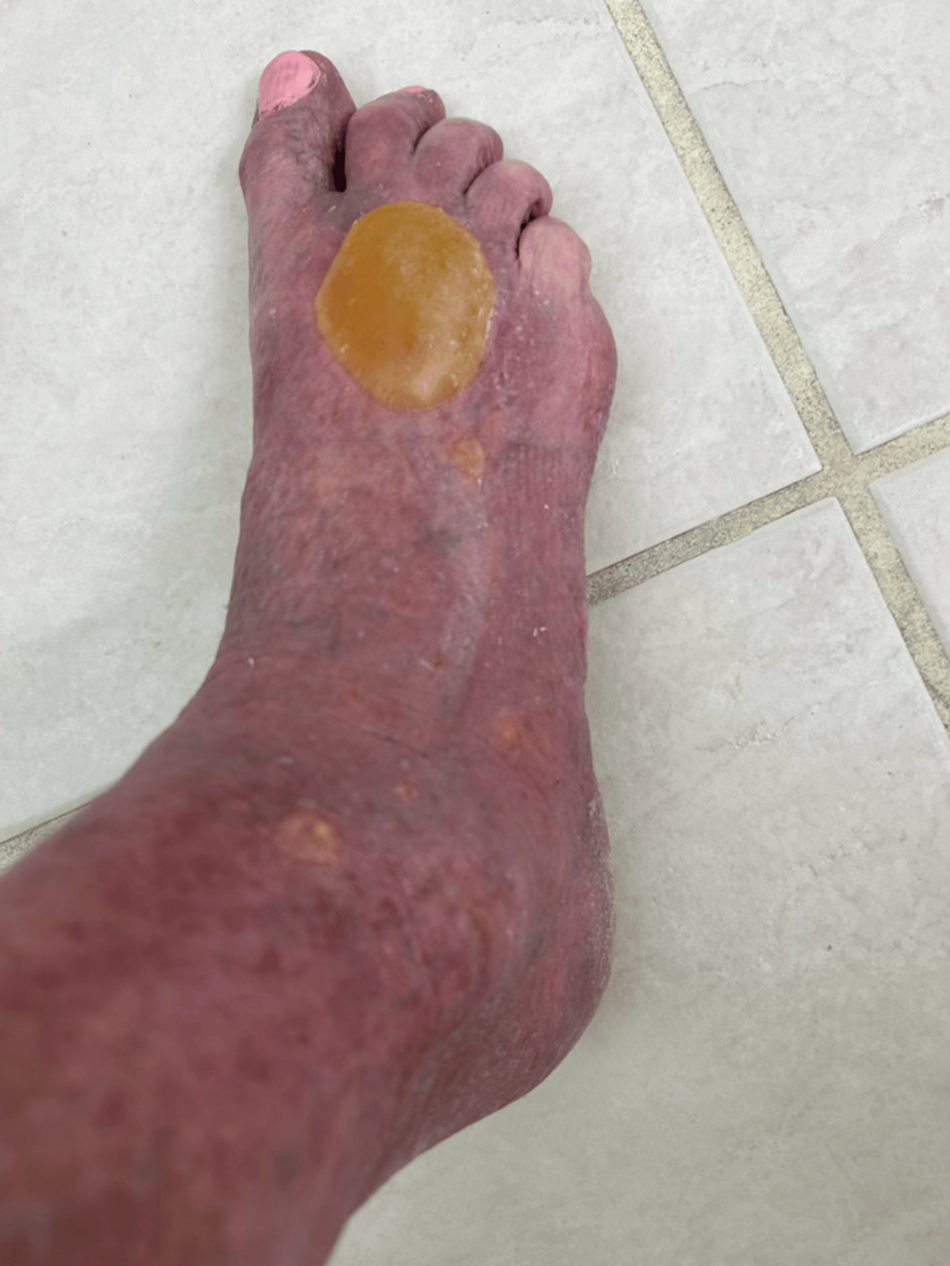
Clinical presentation of bullous pemphigoid on the right foot (week 1); large tense bullae are noted

**Figure 2:**
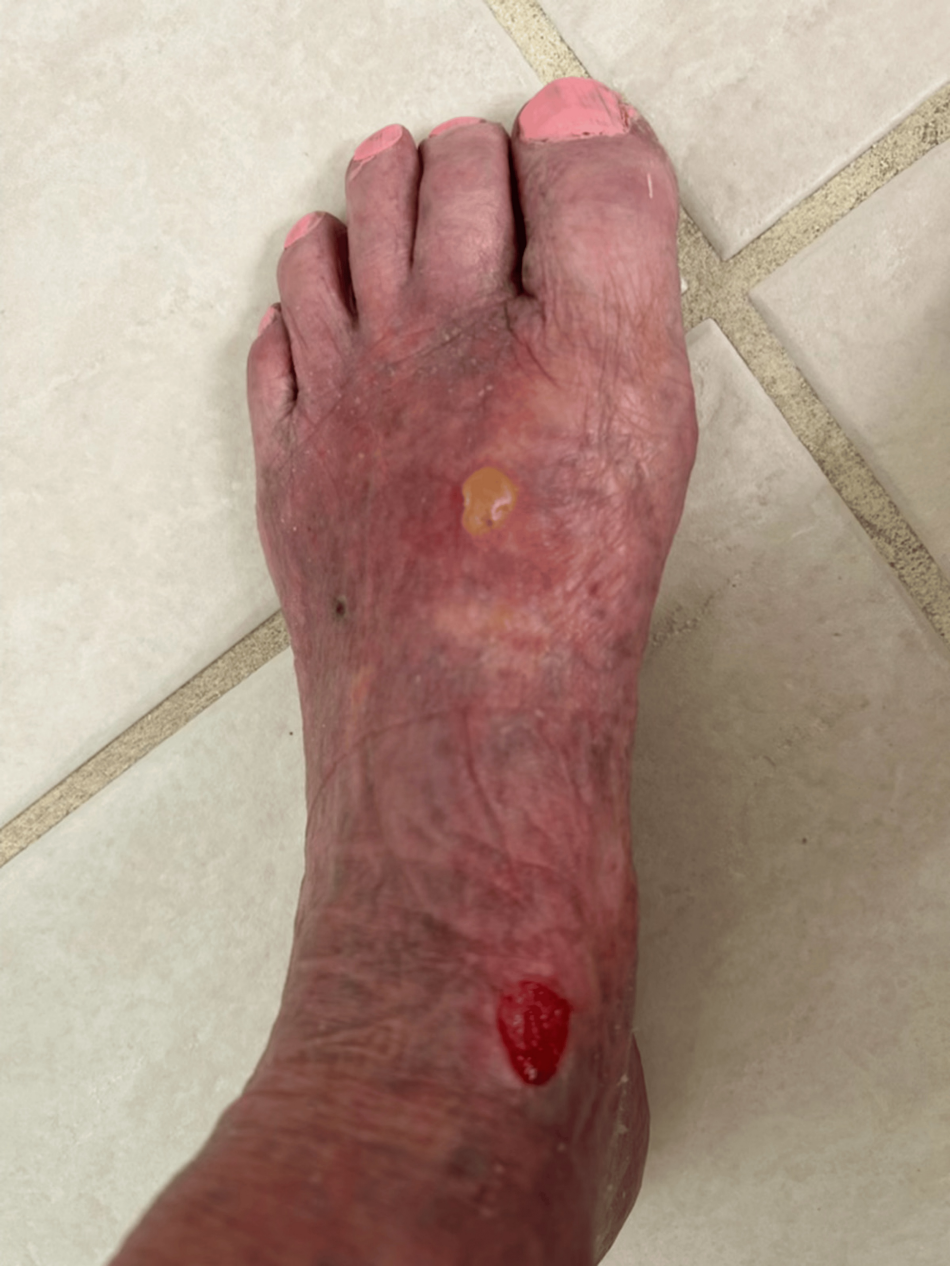
Clinical presentation of bullous pemphigoid on the left foot; healing bullae with erosions are seen

The patient continued to follow up yearly with benign mammogram findings. She returned in April 2024 for a follow-up with a reported development of a blistering rash on both feet (Figure 1 and Figure 2).

The patient reported a rash with multiple bullae on the dorsal sides of both feet, which began in December 2023 and progressively worsened. She went to a dermatologist who performed a 4-millimeter punch biopsy of the lesion. The pathology report demonstrated linear IgG and C3 deposition along the dermal/epidermal junction consistent with bullous pemphigoid. The dermatologist prescribed betamethasone dipropionate topical cream and instructed the patient to apply it twice daily.

On review of the patient’s medications, she was taking cetirizine, mupirocin topical ointment, albuterol hydrofluoroalkane (HFA) inhaler, tiotropium-olodaterol inhaler, bumetanide, losartan, hydroxyzine hydrochloride (HCl), prednisone, pregabalin, levothyroxine, potassium chloride oral liquid, and pravastatin sodium. Notably, the patient had just started on Lyrica (pregabalin) two weeks before the onset of the rash. The patient was instructed to stop Lyrica and continue the application of topical steroid cream. Eighteen weeks after the rash first appeared, the patient showed signs of improvement but had not yet fully recovered.

## Discussion

This case report presents a rare instance of pregabalin-induced BP in a 75-year-old female, highlighting the importance of recognizing drug-induced etiologies in patients presenting with new or unusual dermatologic symptoms. The development of BP following pregabalin initiation is notable due to the relatively infrequent association of this medication with bullous disorders.

While various medications such as diuretics, NSAIDs, antibiotics, and DPP-4 inhibitors have been documented as triggers for BP, cases specifically linking pregabalin to BP are scarce. Upon review, eight cases of cutaneous hypersensitivity reactions caused by pregabalin have been reported [7]. Compared to those reports, this case further contributes to the limited body of evidence of pregabalin-induced BP by providing additional clinical details and emphasizing the importance of considering pregabalin as a potential trigger for BP. 

Several investigations have revealed the genetic susceptibility to BP through an association with specific major histocompatibility complex (MHC) class II alleles. For example, research has shown a significant association with the human leukocyte antigen-DQβ1*0301 allele in populations of European descent. Similarly, in the Japanese population, associations have been identified with the HLA-DRβ1*04, HLA-DRβ1*1101, and HLA-BDB1*0302 alleles [8]. The proposed mechanism by which these HLA alleles contribute to BP susceptibility involves enhancing the presentation of basement membrane zone (BMZ) antigens to T cells. These alleles recognize conserved BMZ antigen epitopes, thereby initiating autoimmunity in BP patients. The pathogenesis of drug-associated BP is thought to originate from the ability of certain drugs to alter the antigenic properties of the immune response. This alteration enables the drugs to bind to molecules within the basement membrane zone, thereby inducing the formation of anti-basement membrane zone antibodies. Another theory suggests that these drugs may structurally modify molecules, uncovering previously hidden epitopes that stimulate an immune response [8]. 

The precise mechanism by which pregabalin induces BP is not fully understood. One theory suggests that pregabalin may alter immune system function, leading to an autoimmune response against the skin's basement membrane, involving autoantibodies against BP180 and BP230, key components of the dermal-epidermal junction [3, 9]. Another possibility is that pregabalin-induced changes in neuronal activity may affect skin integrity or immune surveillance, making the skin more susceptible to autoimmune attacks [2]. Further research is needed to elucidate the precise mechanisms involved.

Recognizing pregabalin as a potential trigger for BP is crucial for healthcare providers. Awareness of this association can lead to prompt identification and appropriate management, preventing complications and improving patient outcomes. Clinicians should maintain a high index of suspicion for drug-induced BP in patients who develop blistering skin conditions while on pregabalin and consider discontinuation of the medication as part of the management strategy [10]. 

The treatment of BP primarily focuses on controlling inflammation, suppressing the autoimmune response, and managing symptoms to improve the patient’s quality of life. Topical corticosteroids, such as clobetasol, are often the first-line therapy, particularly in patients with localized or mild disease, as they reduce inflammation and blister formation [11]. Potent topical corticosteroids have been found to be the most effective treatment during an acute stage of BP. In this case, the use of topical corticosteroids resulted in symptom improvement without the need for further interventions. In some cases, immunosuppressive agents, such as azathioprine, methotrexate, or mycophenolate mofetil, may be added to reduce the need for prolonged high-dose corticosteroid use. In recent years, biologic agents such as rituximab, a monoclonal antibody targeting CD20 on B cells, have shown promise in treating severe or treatment-resistant BP, offering a steroid-sparing option [11]. Additionally, IVIG and omalizumab, an anti-IgE antibody, are emerging therapies that provide alternative mechanisms for controlling BP in difficult-to-treat cases [12].

Future research should focus on exploring the pathophysiological mechanisms underlying pregabalin-induced BP and identify potential risk factors. Studies investigating the molecular and immunological pathways involved could provide deeper insights into how pregabalin triggers this autoimmune response. Targeted research, such as investigating immune modulation in pregabalin users or identifying potential biomarkers for BP onset, would enhance our understanding of the etiology of pregabalin-induced BP. Increased reporting of similar cases will help build a more comprehensive understanding of medication-induced BP, facilitating better diagnosis and management. Understanding the triggers, mechanisms, and at-risk populations will enhance patient safety. Clinical trials or observational studies on larger populations could help validate these findings and guide clinical practice.

## Conclusions

This case highlights pregabalin-induced BP in a 75-year-old female, underscoring the importance of recognizing drug-induced dermatologic conditions. The discontinuation of pregabalin and application of topical corticosteroids led to significant symptom improvement. Clinicians should maintain a high level of suspicion for medication-induced BP, particularly in patients presenting with new blistering disorders while on medications like pregabalin. Increased awareness and prompt identification of such adverse drug reactions can improve patient outcomes. Future studies on pregabalin’s dermatologic side effects will be beneficial in understanding its full spectrum of adverse effects.
